# Highly Dispersed Indium Oxide Nanoparticles Supported on Carbon Nanorods Enabling Efficient Electrochemical CO_2_ Reduction

**DOI:** 10.1002/smsc.202100029

**Published:** 2021-08-03

**Authors:** Binbin Pan, Guotao Yuan, Xuan Zhao, Na Han, Yang Huang, Kun Feng, Chen Cheng, Jun Zhong, Liang Zhang, Yuhang Wang, Yanguang Li

**Affiliations:** ^1^ Institute of Functional Nano & Soft Materials (FUNSOM) Jiangsu Key Laboratory for Carbon-Based Functional Materials and Devices Soochow University Suzhou 215123 China; ^2^ State Key Laboratory for Oxo Synthesis and Selective Oxidation Suzhou Research Institute of LICP Lanzhou Institute of Chemical Physics (LICP) Chinese Academy of Sciences Lanzhou 730000 China; ^3^ Macao Institute of Materials Science and Engineering Macau University of Science and Technology Taipa 999078 Macau SAR China

**Keywords:** carbon nanorods, CO_2_ reduction, flow cell, formate, indium oxide nanoparticles

## Abstract

Indium‐based materials can selectively reduce CO_2_ to formate, but their activities still fall short of expectations to be considered for practical applications. Structural engineering at the nanoscale offers a promising solution. However, it is challenging to directly prepare nanostructures of metallic indium because of its low melting point and high oxophilicity. Herein, a strategy to prepare highly dispersed indium oxide nanoparticles as the precatalyst supported on conductive carbon nanorods from annealing the MIL‐68 (In) precursor is proposed. When assessed in an H‐cell, the product enables CO_2_ reduction to formate with great faradaic efficiency of around 90% over a wide potential window in 0.5 m KHCO_3_. When applied in a gas‐diffusion‐electrode‐based flow cell, the catalyst delivers large current density of up to 300 mA cm^−2^ in 1 m KOH, great formate faradaic efficiency and decent stability. These results indicate the commercial viability of the catalyst even though the carbonate buildup at the gas diffusion electrode remains an issue of future research.

## Graphical abstract

Highly dispersed In_2_O_3_ nanoparticles supported on conductive carbon nanorods are prepared from annealing the MIL‐68 (In) precursor, and investigated as the precatalyst for electrochemical CO_2_ reduction to formate. The product exhibits great formate faradaic efficiency of around 90% over a wide potential window, large current density of up to 300 mA cm^−2^ and decent stability.

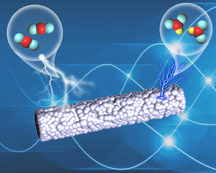

## 1 Introduction

Electrochemical CO_2_ reduction reaction (CO_2_RR) offers an effective strategy to valorize atmospheric CO_2_ to fuels and value‐added chemicals using renewables, and has attracted quickly growing attention over recent years.^[^ ^1^, ^2^, ^3^, ^4^, ^5^ ^]^ Among various CO_2_RR products, formic acid (or formate) is a two‐electron reduction product of great commercial and industry interest.^[^ ^6^, ^7^, ^8^ ^]^ It is a promising hydrogen carrier and is used as the chemical fuel for formic acid fuel cells.^[^ ^9^, ^10^, ^11^ ^]^ Based on the latest techno‐economic analysis, selectively reducing CO_2_ to formic acid could be the most commercially profitable approach among all CO_2_RR pathways when a high‐productivity and high‐selectivity process is achieved.^[^ ^12^, ^13^ ^]^ Unfortunately, electrocatalysts currently available for the CO_2_‐to‐formate upgrading are still limited by unsatisfactory reaction activities. Their partial current density remains below 100 mA cm^−2^ in most cases.^[^ ^14^, ^15^, ^16^, ^17^, ^18^, ^19^, ^20^, ^21^ ^]^ It is therefore of practical significance to develop efficient electrocatalyst materials and electrochemical devices that can enable the conversion of CO_2_ to formic acid at current densities of commercial relevance (>200 mA cm^−2^).

Indium (In) is known to catalyze CO_2_RR to formate selectively.^[^ ^22^ ^]^ Excellent faradaic efficiency toward formate (>90%) has been achieved in literatures.^[^ ^23^, ^24^, ^25^, ^26^, ^27^ ^]^ Albeit with recent advances, it remains challenging to devise In‐based catalysts having high formate partial current density without compromise to selectivity. To this end, designing nanostructured catalysts with high‐density and undercoordinated active sites is highly desirable. However, the low melting point and proneness to oxidation of metallic In renders difficult the direct preparation of its nanostructures. An alternative strategy is to prepare In compounds (such as oxides, sulfides, and so on) with a precise control over the size and shape as the precatalysts, and in situ convert them to metallic In nanostructures (which often inherit the structural feature of original precatalysts) for CO_2_RR. Metal organic frameworks (MOFs) consist of assembled metal nodes and organic struts, and have large specific surface areas and abundant porosity.^[^ ^28^, ^29^ ^]^ High‐temperature pyrolysis converts them to corresponding metal/metal oxide nanoparticles supported on carbonaceous supports, which have been widely investigated for a range of electrochemical applications.^[^ ^30^, ^31^, ^32^ ^]^ This approach, however, has not be pursued for In‐based materials for CO_2_RR in our best knowledge.

Herein, we present a catalyst processing strategy that allows for the CO_2_‐to‐formate conversion at high current density and high faradaic efficiency. We use a metal‐organic framework MIL‐68 (In) as the precursor to synthesize highly dispersed In_2_O_3_ nanoparticles supported on carbon nanorods (In_2_O_3_@CNR). The resultant product enables great formate faradaic efficiency of around 90% across a wide potential window in our H‐cell measurements, and large current density up to 300 mA cm^−2^ without compromise to selectivity and stability in our flow cell measurements.

## 2 Results and Discussion

**Figure 1:**
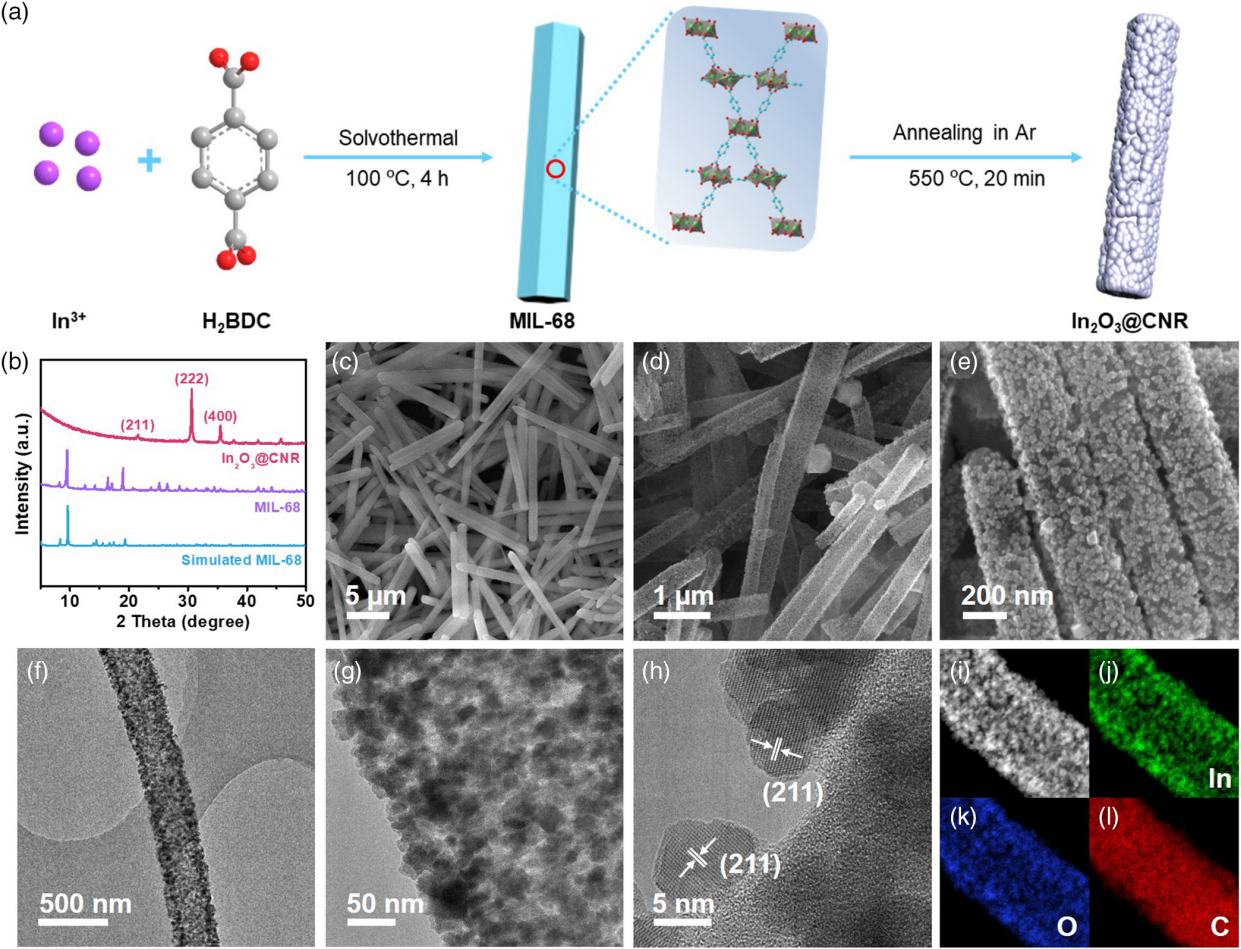
Preparation and structural characterizations of In_2_O_3_@CNR. a) Schematic synthetic procedure of In_2_O_3_@CNR; b) XRD patterns of MIL‐68 (In) and In_2_O_3_@CNR; c) SEM image of MIL‐68; d,e) SEM images and f–h) TEM images of In_2_O_3_@CNR at different magnifications; i) STEM image and j–l) corresponding EDS elemental mapping of In, O, C in In_2_O_3_@CNR.

We began by preparing In_2_O_3_@CNR via a two‐step procedure, as schematically shown in **Figure** **1**a. MIL‐68 (In) MOF was first synthesized from the reaction between In^3+^ ions and terephthalic acid in *N,N*‐dimethylformamide (DMF) following a previous report.^[^ ^33^ ^]^ The XRD pattern of this intermediate product supports the formation of the desired crystalline MOF structure (Figure 1b). Under scanning electron microscopy (SEM), MIL‐68 (In) is revealed to have a 1D rod shape with smooth surfaces, length of 10–15 μm and width of 200–300 nm (Figure 1c). After the second annealing step at 550 °C, the organic skeleton of MIL‐68 (In) becomes carbonized and transforms to carbon nanorods of comparable dimensions, while the In^3+^ ions in MIL‐68 (In) are converted to In_2_O_3_ nanoparticles. The X‐ray diffraction (XRD) pattern of In_2_O_3_@CNR shows diffraction peaks assignable to the cubic In_2_O_3_ phase (Figure 1b). SEM and transmission electron microscopy (TEM) imaging reveals that these In_2_O_3_ nanoparticles have a size of 5–20 nm, and are uniformly dispersed on the carbon rod support (Figure 1d–g and Figure S1, Supporting Information). They are formed from the coalescence of mobile In species during annealing that phase segregate and crystallize on the surface of carbon nanorods instead of remaining buried inside as isolated atoms. Distinct lattice fringes corresponding to the (211) plane of In_2_O_3_ are observed from high‐resolution TEM (Figure 1h). The uniform spatial distribution of In, O, and C elements are also verified by energy dispersive spectroscopy (EDS) elemental mapping under scanning transmission electron microscopy (STEM) (Figure 1i–l). Moreover, thermal gravimetric analysis (TGA) suggests that the In_2_O_3_ weight percentage in In_2_O_3_@CNR is 75.1 wt% (Figure S2, Supporting Information). N_2_ adsorption–desorption measurement reveals that the product has a BET surface area of 210 m^2^ g^−1^ (Figure S3, Supporting Information). The large specific surface area of In_2_O_3_@CNR would provide high‐density active sites conducive to efficient CO_2_RR. We additionally investigate the influence of the annealing temperature on the product morphology, and find that the increased annealing temperature results in growing In_2_O_3_ nanoparticle size and increasing surface roughness (Figure S4, Supporting Information).

**Figure 2:**
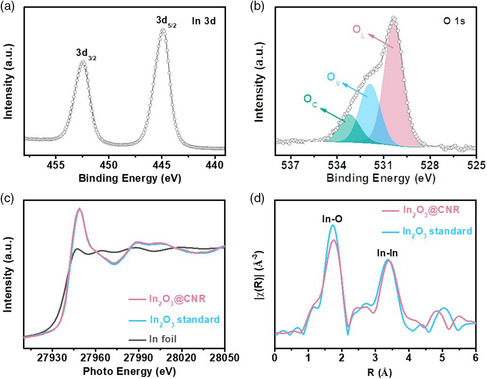
Spectroscopic characterizations of In_2_O_3_@CNR. a) In 3d XPS and b) O 1s XPS spectra of In_2_O_3_@CNR; c) XANES and d) corresponding Fourier‐transformed EXAFS spectra of In_2_O_3_@CNR in comparison with the In foil and In_2_O_3_ standards.

The bonding configuration and electronic structure of In_2_O_3_@CNR were further interrogated using X‐ray photoelectron spectroscopy (XPS) and X‐ray absorption spectroscopy (XAS). The deconvolution of its In 3d XPS spectrum uncovers the dominant contribution of In^3+^ with 3d_2/5_ and 3d_3/2_ located at 444.9 and 452.4 eV, respectively (**Figure** **2**a).^[^ ^34^ ^]^ The peaks centered at 530.3, 531.8, and 533.2 eV in the O 1s spectrum arise from the lattice oxygen of In_2_O_3_ (O_L_), O‐vacancies (O_V_), and chemisorbed oxygen species (O_C_), respectively (Figure 2b).^[^ ^34^, ^35^, ^36^ ^]^ X‐ray absorption near‐edge structure (XANES) at the In K edge is consistent with the In 3d XPS (Figure 2c), and suggests that In in In_2_O_3_@CNR is mainly in the trivalent state. In—In and In—O bonds are clearly revealed from the corresponding Fourier‐transformed extended X‐ray absorption fine structure (EXAFS) spectrum (Figure 2d).

**Figure 3:**
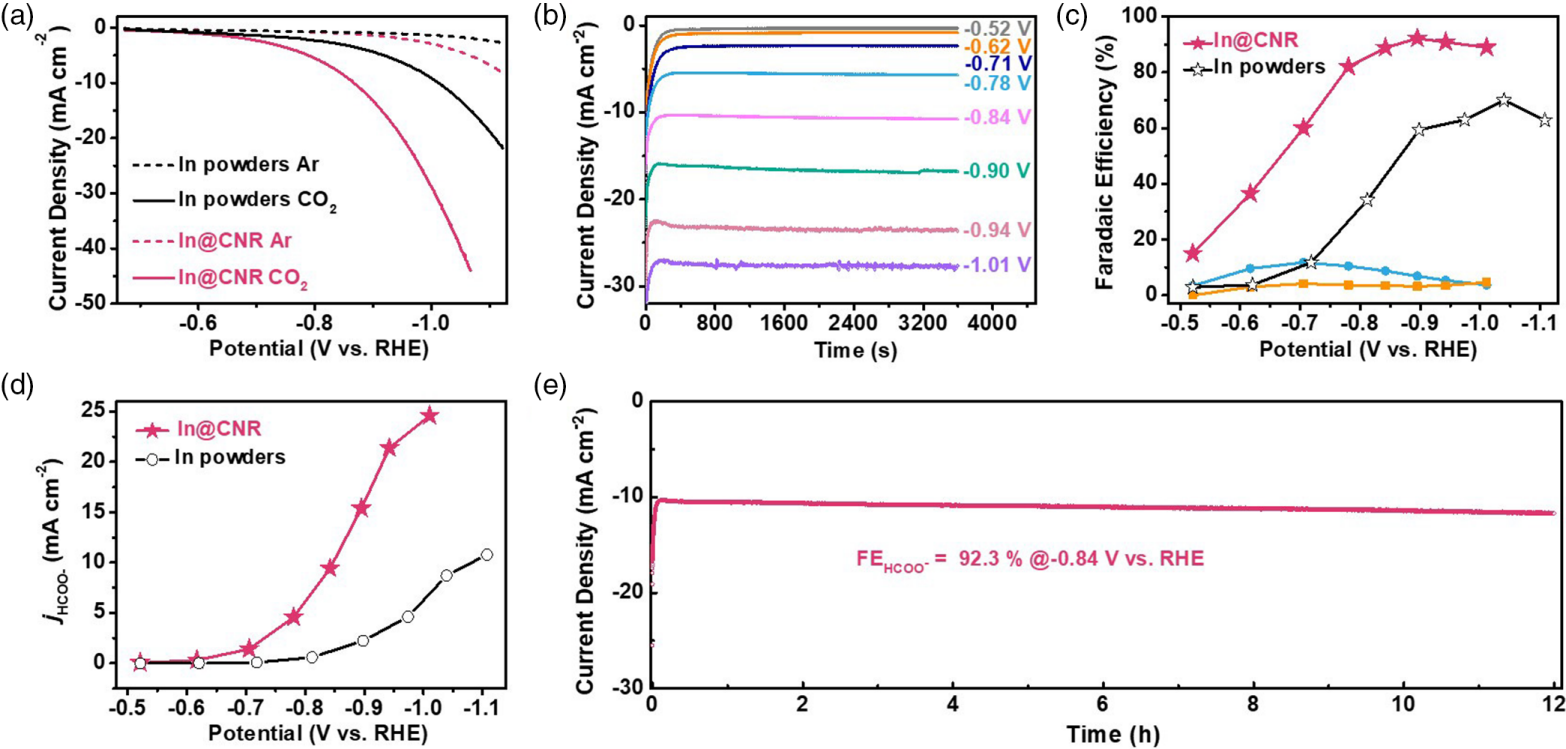
CO_2_RR performance of In@CNR in the H‐cell. a) Polarization curves of In@CNR and commercial In powders in Ar‐ or CO_2_‐saturated 0.5 m KHCO_3_; b) chronoamperometric curves of In@CNR at a few different potentials; c) faradaic efficiency for formate, CO or H_2_ on In@CNR and In powders; d) formate partial current density of In@CNR and In powders; e) long‐term chronoamperometric stability of In@CNR at −0.84 V.

We next evaluated the electrocatalytic performance of In_2_O_3_@CNR for CO_2_RR to formate in an H‐cell filled with 0.5 m KHCO_3_. Please note that In_2_O_3_ here serves as the precatalyst, and would transform to metallic In (verified by our XRD analysis) as the real catalyst for CO_2_RR,^[^ ^7^ ^]^ which is accordingly denoted as In@CNR (Figure S5, Supporting Information). Commercial In powders are introduced as a control for comparison side by side. Their SEM characterization is available from Figure S6, Supporting Information. When the electrolyte is saturated with Ar, the polarization curves of In@CNR and In powders are exclusively contributed from H_2_ evolution (**Figure** **3**a). When the electrolyte is saturated with CO_2_, the cathodic current density increases markedly due to CO_2_RR. The current density of In@CNR is much larger than that of commercial In powders. For instance, at −1.0 V (vs reversible hydrogen electrode or RHE, the same hereinafter), In@CNR delivers the current density of 28.9 mA cm^−2^, about three times larger than that of the control sample (9.1 mA cm^−2^).

To quantify CO_2_RR products, we carried out chronoamperometric (*i*–*t*) measurements of In@CNR in the potential range from −0.52 to −1.01 V each for 1 h (Figure 3b). Chronoamperometric data of In powders are summarized in Figure S7, Supporting Information. For In@CNR, formate overwhelmingly dominates the product distribution across the potential window (Figure 3c). The faradaic efficiency for both CO and H_2_ is below 10%. The faradaic efficiency for formate plateaus at 90% in the potential window of −0.8 to −1.0 V. By contrast, the highest formate faradaic efficiency of In powders is only around 70%. The formate partial current density of In@CNR reaches a remarkable current density of 25 mA cm^−2^ at −1.01 V, far exceeding that of In powders (Figure 3d). The stark difference unambiguously demonstrates the excellent activity of our In@CNR for CO_2_RR. The high activity and selectivity toward formate may stem from the large surface area of In@CNR that inherits the nanoscale morphology of In_2_O_3_@CNR and consequently provides abundant active sites compared with the control. Small particle size may also give rise to the increasing coordinative unsaturation of surface atoms or other defects that are known to promote the site‐specific activity. Compared to the previous reports about In‐based catalysts,^[^ ^37^, ^38^, ^39^, ^40^ ^]^ the enhanced formate partial current density of our In@CNR translates to an increase in the activity (Table S1, Supporting Information). For example, the Hou and coworkers reported that dendritic In foams from electrodeposition could convert CO_2_ to formate at −0.86 V with faradaic efficiency of 86%. The corresponding current density was only 5.8 mA cm^−2^.^[^ ^39^ ^]^ The performance metric we achieve here is also comparable with the best prior results under similar testing conditions.^[^ ^23^ ^]^ In addition, it is found that products from lower or higher annealing temperature at the second steps all exhibit inferior formate selectivity and partial current density (Figure S8, Supporting Information). We also carried out long‐term testing by biasing In@CNR at −0.86 V for 12 h (Figure 3e). Its cathodic current density maintains at around 11 mA cm^−2^ during the entire course of the evaluation. The average faradaic efficiency is measured to be 92.3% at the end of electrolysis.

**Figure 4:**
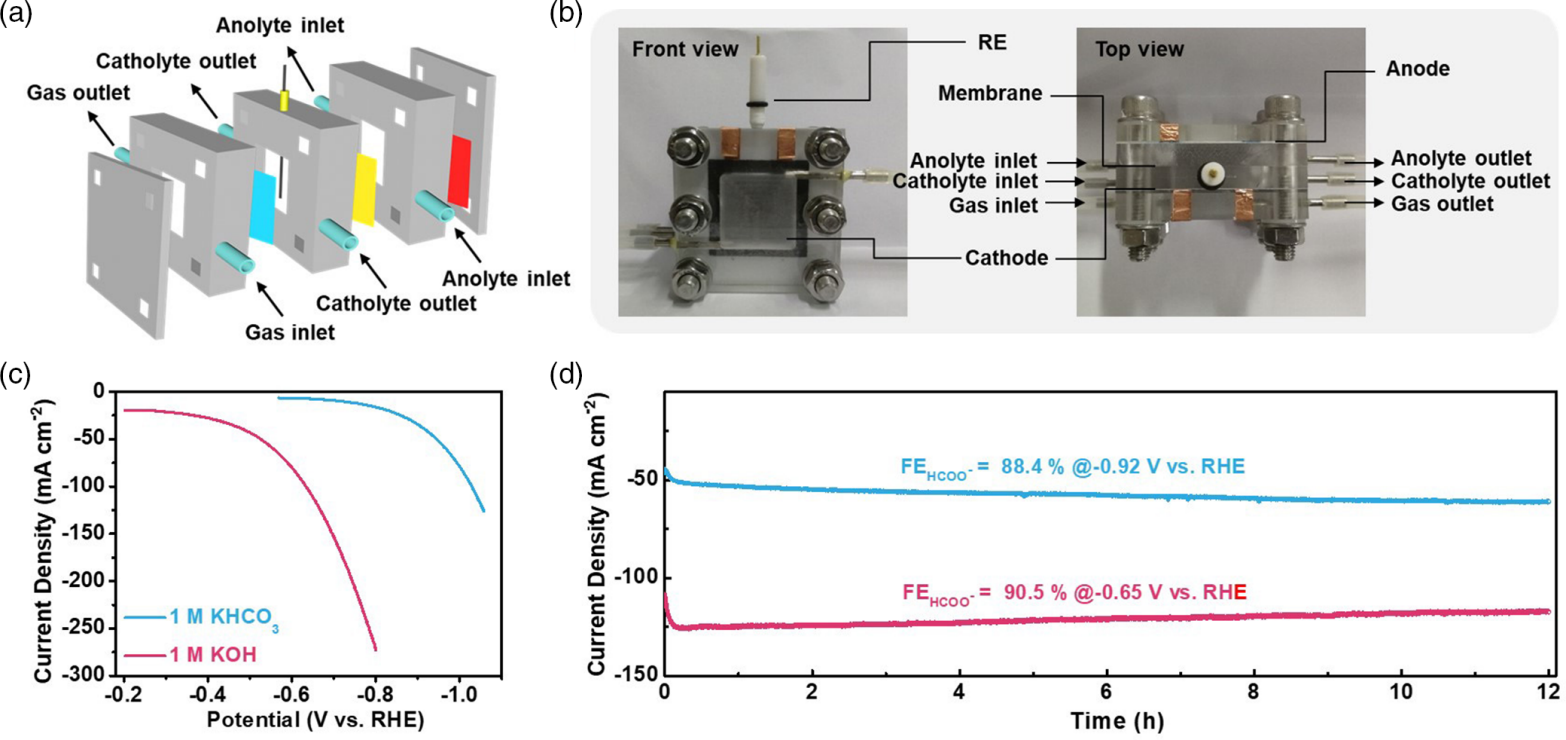
CO_2_RR performance of In@CNR in the flow cell. a) Schematic illustration of the flow cell configuration; b) pictures of the flow cell viewed from the front and the top showing its different components; c) polarization curves of In@CNR in 1 m KHCO_3_ and 1 m KOH; d) long‐term chronoamperometric stability of In@CNR at −0.92 V in 1 m KHCO_3_ and at −0.65 V in 1 m KOH.

We further pursued to scale up the formate production from CO_2_RR based on the gas diffusion electrode (GDE) configuration. The utilization of GDE enables the direct reaction of CO_2_ gas at the triple‐phase boundaries among catalysts, electrolytes and reactants. It overcomes the current limitation imposed by the low solubility and diffusivity of CO_2_ in aqueous solution in conventional H‐cells. We fabricated the In@CNR GDE and conducted CO_2_RR tests in a customized flow cell reactor, as shown in **Figure** **4**a,b. The CO_2_RR activity of In@CNR was investigated in both 1 m KHCO_3_ and 1 m KOH. In 1 m KHCO_3_, the onset potential of In@CNR agrees with the value measured in the H‐cell, whereas its current density (80 mA cm^−2^) markedly increases by nearly twofolds compared with that in H‐cell (28 mA cm^−2^) at −1.0 V as a result of the enhanced mass transfer through GDE (Figure 4c). In 1 m KOH, the onset potential of In@CNR is improved to around −0.3 V as the electrochemical formate production is known to be strongly favored at high pHs.^[^ ^7^, ^41^ ^]^ Most remarkably, its current density quickly rises to 275 mA cm^−2^ at −0.8 V, which substantially exceeds the threshold value (200 mA cm^−2^) to be considered for future commercialization.

The long‐term stability of In@CNR was examined at high current densities in 12‐h operations (Figure 4d). We confirm decent operating stability with current density of around 60 mA cm^−2^ at −0.86 V in 1 m KHCO_3_ and of around 120 mA cm^−2^ at −0.65 V in 1 m KOH. The average formate faradaic efficiency is determined to be 88.4% and 90.5%, respectively. Note that the slight increase in current density (about 20% increase) during the long‐term electrolysis in 1 m KHCO_3_ is believed to result from a slow activation process presumably via catalyst surface roughening, while the slight decrease in current density (6% decrease) in 1 m KOH is caused by the carbonate buildup at the GDE from the electrolyte carbonation. The corresponding formate partial current density is 108 mA cm^−2^ at −0.65 V in 1 m KOH with the calculated cathodic energy efficiency of about 50%, indicating an increase in the activity relative to the best prior studies using In‐based catalysts^[^ ^24^, ^25^ ^]^ except for InP quantum dots reported by the Sargent group.^[^ ^41^ ^]^ SEM characterization of the post‐use catalyst shows that the majority of nanoparticles remain on the carbon naorod support even though they appear to be smaller after the reduction and activation (Figure S9, Supporting Information).

It should be admitted at this stage that the catalyst lifespan in our work is far from meeting the demanding needs for commercialization. We encounter the GDE flooding issue that compromises the operating lifetime. We also observe the carbonate formation via the reaction between CO_2_ and the electrolyte (in particular alkaline electrolytes). As shown in Figure S10, Supporting Information, both the front side and back side of the spent GDE contain white powder residues that are analyzed to be carbonate by XRD. The carbonate buildup is a general challenge to flow cell or membrane electrode assembly (MEA) measurements in alkaline solution.^[^ ^42^, ^43^, ^44^ ^]^ It not only leads to the loss of GDE's super‐hydrophobicity and causes the GDE flooding, but also clogs the pores and prohibits the CO_2_ diffusion through GDE. Electrolyzer engineering tackling with the above roadblocks for practical applications would be one of the future directions for CO_2_RR.

## 3 Conclusion

In summary, we demonstrated the preparation of In_2_O_3_@CNR derived from MIL‐68 (In) as the precatalyst for efficient CO_2_RR to formate. The product features nanosized In_2_O_3_ nanoparticles uniformly dispersed on carbon nanorods. Under cathodic potentials, In_2_O_3_ is transformed to metallic In as the working catalyst for CO_2_RR. When assessed in an H‐cell, our In@CNR enabled the selective formate production with great faradaic efficiency of 90% over a wide potential window in CO_2_‐satured 0.5 m KHCO_3_. When assessed in a GDE‐based flow cell, our catalyst achieved large current density up to 300 mA cm^−2^, selectivity of >90%, cathodic energy efficiency of about 50% and operation stability of >12 h in 1 m KOH. The operating lifetime and CO_2_ single‐pass utilization might be improved in the future by electrolyzer engineering so as to retard the GDE flooding and carbonate formation.

## Conflict of Interest

The authors declare no conflict of interest.

## Data Availability Statement

The data that support the findings of this study are available from the corresponding author upon reasonable request.

## Supporting information

Supplementary Material
