# Supplementary material for: Highly Dispersed Indium Oxide Nanoparticles Supported on Carbon Nanorods Enabling Efficient Electrochemical CO2 Reduction
Source: Small Sci. 2021 Aug 3;1(10):2100029. doi: 10.1002/smsc.202100029 (PMC11935809; doi:10.1002/smsc.202100029)
Supplement: Supplementary file 1 — Supplementary Material [file SMSC-1-2100029-s001.pdf]

Supporting Information

Highly Dispersed Indium Oxide Nanoparticles Supported on Carbon Nanorods Enabling Efficient Electrochemical CO_2 Reduction

Binbin Pan, Guotao Yuan, Xuan Zhao, Na Han, Yang Huang, Kun Feng, Chen Cheng, Jun Zhong, Liang Zhang, Yuhang Wang and Yanguang Li**

Experimental Section

1. Preparation of In_2O_3@CNR. For the synthesis of MIL-68 (In), 0.5 mmol of 1,4-benzenedicarboxylic acid ($C_8H_6O_4$, Aldrich, 98%) and 0.5 mmol of indium nitrate hydrate ($In(NO_3)_3 \cdot xH_2O$, J&K Scientific, 99.99%) were first dissolved in 10 mL of DMF assisted by ultrasonication for 10 min. The solution was solvothermally reacted in a 25 ml Teflon-lined steel autoclave at 100 °C for 4 h. At the end of the reaction, resultant white solids were collected by centrifugation, washed with DMF and anhydrous ethanol each for three times, and finally vacuum-dried at 70 °C for 12 h. MIL-68 (In) was subsequently annealed at 550 °C under Ar for 20 min to yield the final product In_2O_3@CNR.

2. Materials characterizations. XRD patterns were collected on a PANalytical X-ray diffractometer. XPS analysis was carried out on a Thermo Scientific NEXSA XPS spectrometer. SEM images were taken on a Supera 55 Zeiss scanning electron microscope. TEM and STEM images were taken on a FEI Tecnai F20 transmission electron microscope. TGA was conducted on a Mettler Toledo TGA/DSC1 thermal analyzer in air. Gas sorption experiment was carried out using a Micromeritics 3Flex surface characterization analyzer. XAS was performed at the Shanghai Synchrotron Radiation Facility (SSRF, 14W) and the Taiwan Light Source (TLS, 01C1) beamline. The energy scale of the In K-edge was calibrated against the In foil standard.

3. CO_2RR measurements in the H-cell. To prepare working electrodes, 1 mg of the catalyst powders (In_2O_3@CNR or commercial In powders) and 0.5 mg of Ketjenblack carbon were first dispersed in 200 μL of ethanol, 50 μL of distilled water and 8 μL of 5 wt% Nafion solution, and subjected to vigorous ultrasonication for about 30 min. This catalyst ink was then dropcast onto a 1 × 1 cm^2 carbon fiber paper (Avcarb P75T, Fuel Cell Store) to reach a catalyst areal loading of ~1 mg cm^{-2}. Electrochemical CO_2RR measurements were carried out in an airtight two-compartment H-cell with a graphite rod counter electrode housed in the anodic compartment, and a saturated calomel reference electrode (SCE) and the working

electrode housed in the cathodic compartment. These two compartments were separated by a piece of Nafion exchange membrane and filled with 0.5 M $KHCO_3$ as the electrolyte. During CO_2RR measurements, the catholyte was continuously bubbled with CO_2 at 20 sccm to maintain its saturation. All the potential readings were measured against SCE and then converted to RHE with necessary iR compensation. Gaseous reduction products were analyzed using an on-line gas chromatograph (GC, Aligent 7890B). Formate in the catholyte was quantified by an ion chromatograph (Dionex ICS-600) following the protocol described in our previous publications (*Nat. Commun.* **2018**, *9*, 1320.).

4. CO_2RR measurements in the flow cell. Flow cell measurements were conducted in our custom-built flow cell reactor, which was assembled from the sequential stacking of a gas chamber, a catalyst-loaded GDE cathode, a catholyte chamber (where a Ag/AgCl reference electrode locates), a Nafion exchange membrane, an anolyte chamber and an IrO_2-loaded Ti plate anode (Figure 4b). The volume of both the cathode and anode chambers was ~7.2 cm^3. During CO_2RR measurements, 30 sccm of CO_2 was continuously fed to the gas chamber. The electrolyte (1 M $KHCO_3$ or 1 M KOH) was pumped to circulate through the catholyte and anolyte chambers at the rate of 5 ml min^{-1} by a peristaltic pump. The cathodic energy efficiency of formate was calculated as follows (*Electrochim. Acta* **2014**, *141*, 216; *J. Am. Chem. Soc.* **2020**, *142*, 5702.):

$$EE(\%) = \frac{(1.23\ V - E^0) \times FE}{1.23\ V - E}$$

where E^o is the equilibrium potential of CO_2RR to formate (+0.19 V versus RHE in 1 M KOH), *FE* is the faradaic efficiency of formate, and *E* is the applied potential after 90% ohmic loss compensation.

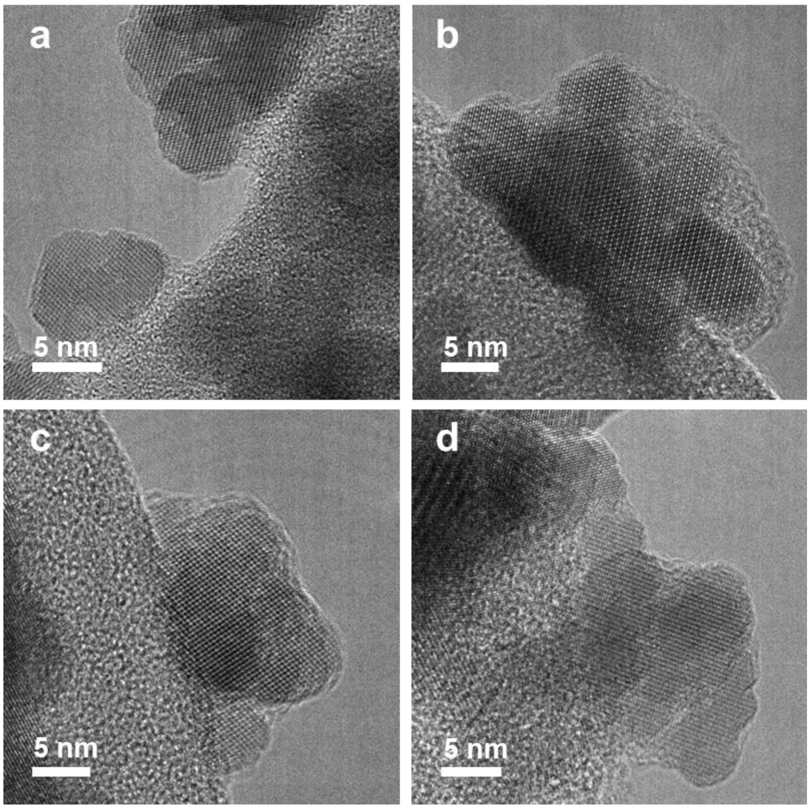

Figure S1. Additional high-resolution TEM images of In_2O_3@CNR.

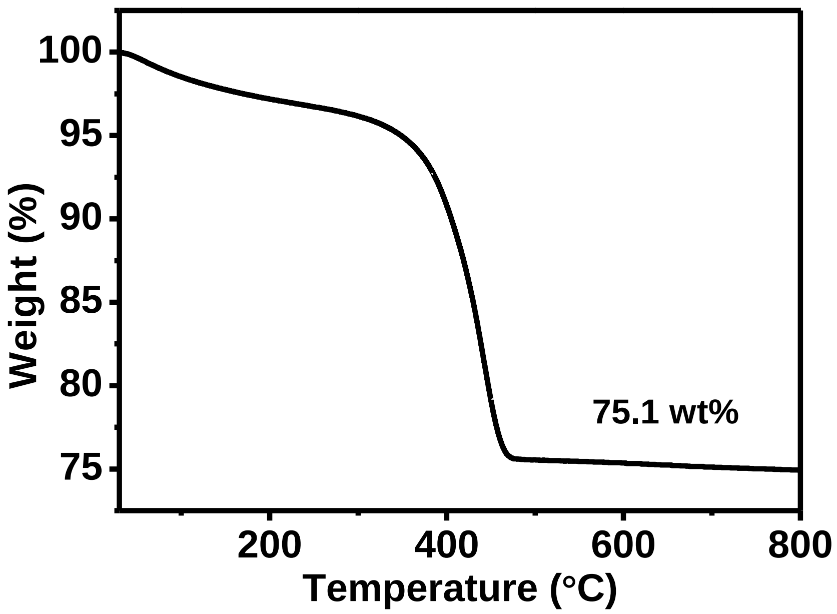

Figure S2. TGA curve of In_2O_3@CNR in air.

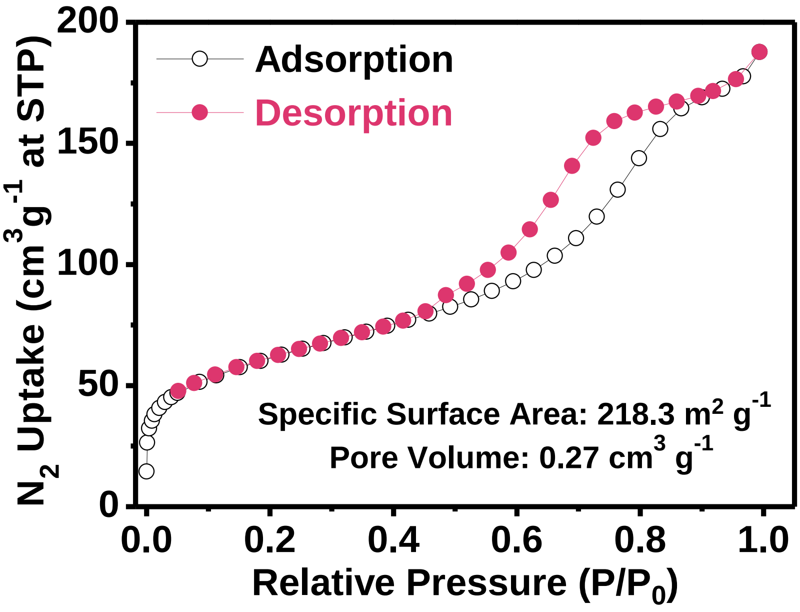

Figure S3. N_2 adsorption-desorption isotherm of In_2O_3@CNR.

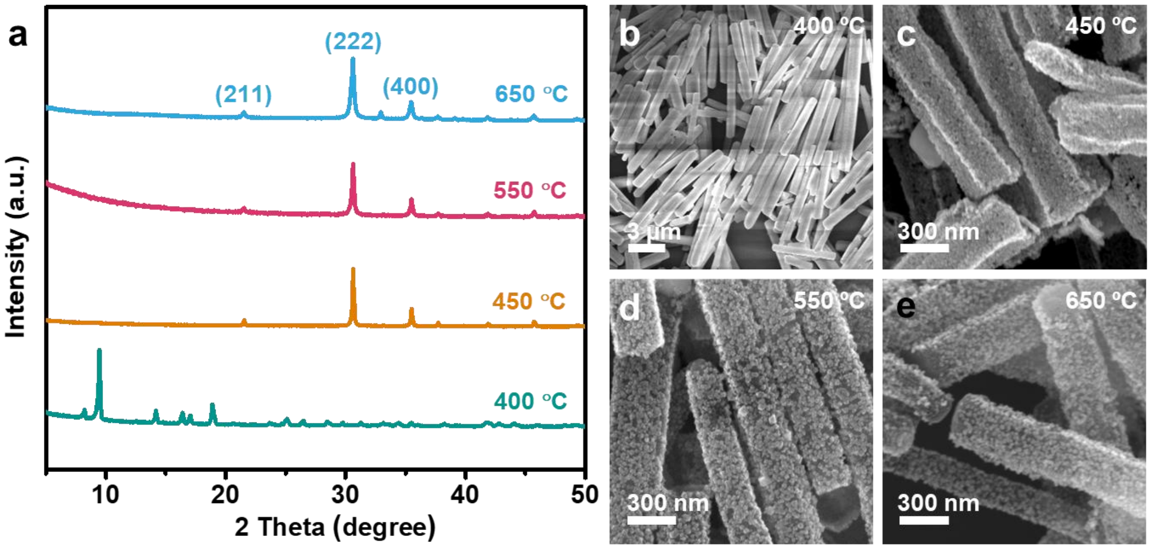

Figure S4. (a) XRD patterns and (b-e) SEM images of the products obtained from different annealing temperatures as indicated.

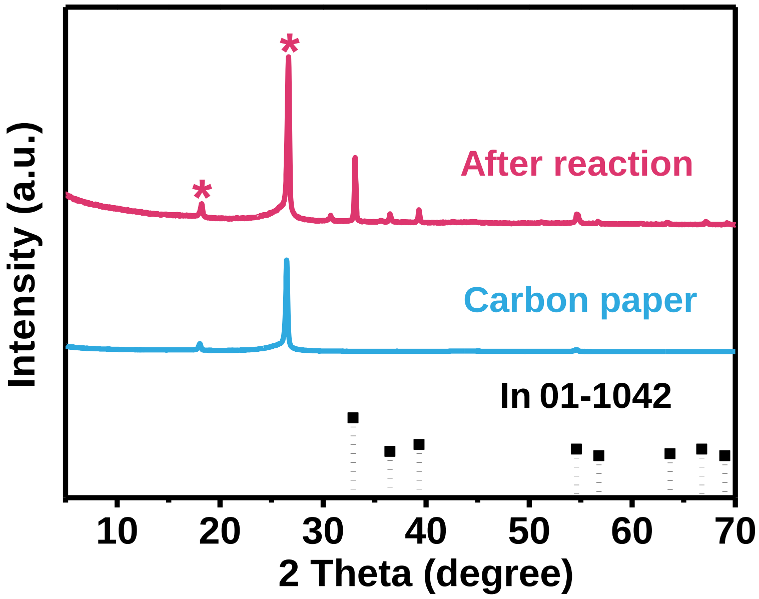

Figure S5. XRD pattern of In_2O_3@CNR after the long-term chronoamperometric stability test, the asterisk indicates the signals of carbon paper.

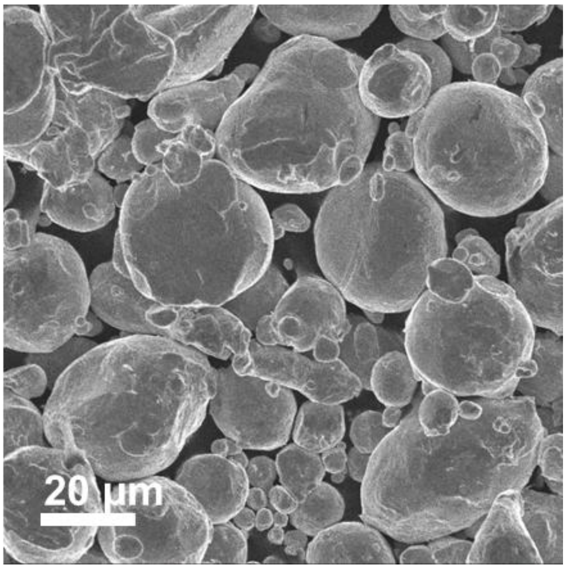

Figure S6. SEM image of commercial In powders.

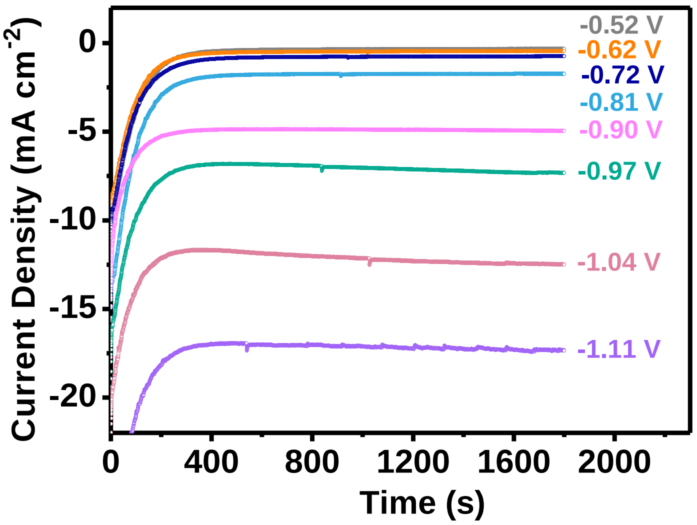

Figure S7. Chronoamperometric curves of commercial In powders at a few selected potentials as indicated.

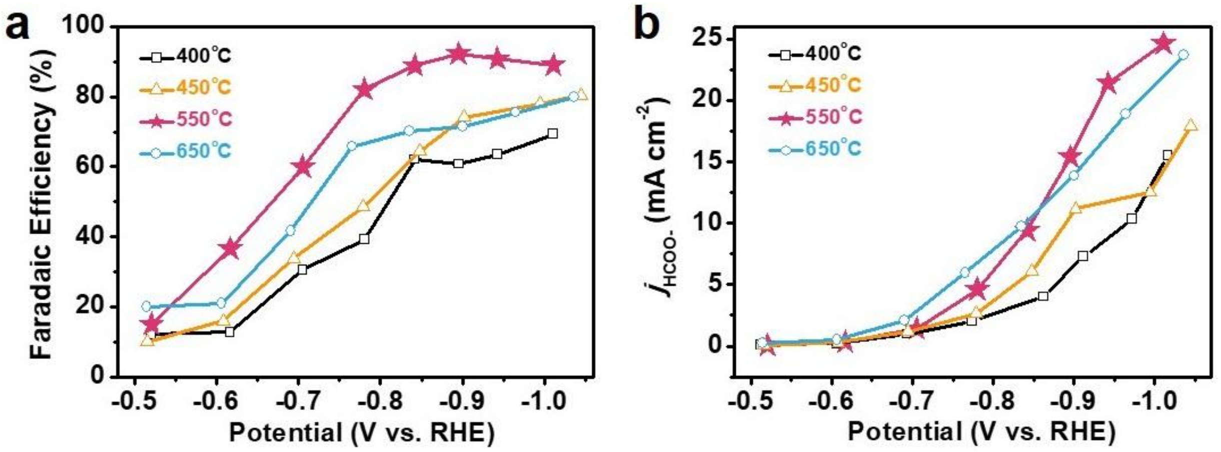

Figure S8. (a) Formate Faradaic efficiency and (b) formate partial current density of products annealed at 400 ºC, 450 ºC, 550 ºC and 650 ºC at the second step. These results can be rationalized by the facts that MOFs cannot be fully carbonized and converted to conductive carbon nanorods at temperatures below 500 ^{o}C, while increasing the annealing temperature beyond 600 ^{o}C leads to larger particle sizes that compromise the CO_2RR performance.

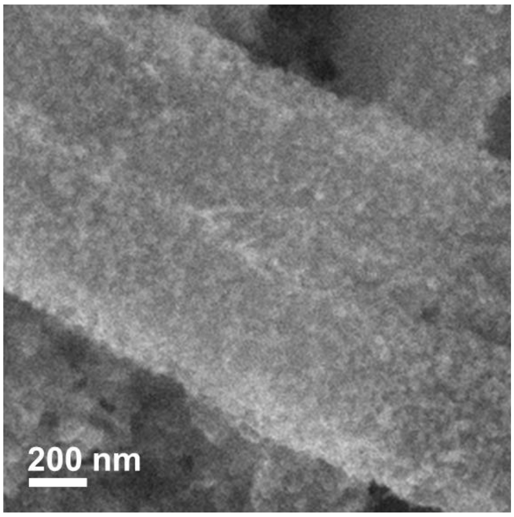

Figure S9. SEM image of the washed catalyst after the long-term chronoamperometric stability test in the flow cell.

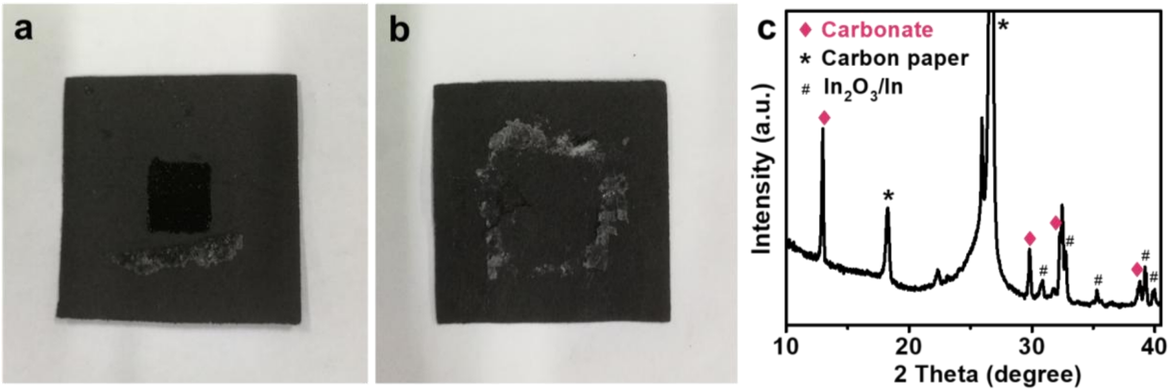

Figure S10. (a,b) Photos of spent GDE viewed from (a) the front side (facing the electrolyte) and (b) the back side (facing the feed gas) showing white powder residues after the long-term chronoamperometric stability test in KOH; (c) XRD pattern of the spent GDE revealing the presence of carbonate from the electrolyte carbonation.

Table S1. Comparison of the CO_2RR-to-formate performances of our In_2O_3@CNR and earlier representative works from literatures measured in H-cells.

**Electroatalysts**	**Electrolyte**	**Formate FE_{max}**	**J_{HCOO^-} @FE_{max}**	**Reference**
In_2O_3@CNR	0.5 M $KHCO_3$	92.3% @ -0.84 V vs. RHE	10.2 mA/cm^2	This work
Indium dendritic foams	0.5 M $KHCO_3$	86% @ -0.86 V vs. RHE	5.8 mA/cm^2	*ChemElectroChem* **2018**, *5*, 253.
H-InO_x NRs	0.5 M $NaHCO_3$	91.7% @ -0.7 V vs. RHE	5 mA/cm^2	*Angew. Chem. Int. Ed.* **2019**, *58*, 5609.
Single-atom Indium$^{\delta+}$–N_4	0.5 M $KHCO_3$	96% @ -0.65 V vs. RHE	8.87 mA/cm^2	*Angew. Chem. Int. Ed.* **2020**, *59*, 22465.
Anodized In	0.5 M K_2SO_4	87.2% @ -1.7 V vs. SCE	5.28 mA/cm^2	*ACS Catal.* **2016**, *6*, 7824.
O-vacancy In_2O_3	0.1 M $KHCO_3$	93.5% @ -0.9 V vs. RHE	14 mA/cm^2	*Sustainable Energy Fuels* **2020**, *4*, 3726.
S-In_2O_3 derived In	0.5 M $KHCO_3$	95% @ -0.98 V vs. RHE	58.9 mA/cm^2	*Nat. Commun.* **2019**, *10*, 892.
